# MRI outcome evaluation in patients with IB2 and IIA2 squamous cervical cancer stages: preliminary results

**DOI:** 10.1186/s13244-022-01269-6

**Published:** 2022-09-16

**Authors:** Qingling Song, Huiting Pang, Rui Tong, Yanmei Zhu, Yahong Luo, Tao Yu, Fan Liu, Yue Dong

**Affiliations:** 1 Graduate School of Dalian Medical University, Dalian, 116044 Liaoning China; 2 Department of Radiology, Cancer Hospital of China Medical University, Cancer Hospital of Dalian University of Technology, Liaoning Cancer Hospital & Institute, Shenyang, 110042 Liaoning China; 3 Department of Gynecology, Cancer Hospital of China Medical University, Cancer Hospital of Dalian University of Technology, Liaoning Cancer Hospital & Institute, Shenyang, 110042 Liaoning China; 4 Department of Pathology, Cancer Hospital of China Medical University, Cancer Hospital of Dalian University of Technology, Liaoning Cancer Hospital & Institute, Shenyang, 110042 Liaoning China

**Keywords:** MRI, Squamous cervical cancer, Disease-free survival, Neoadjuvant therapy, Concurrent chemoradiotherapy

## Abstract

**Objectives:**

To evaluate the therapeutic effect of neoadjuvant therapy (NAT) followed by radical hysterectomy and concurrent chemoradiotherapy (CCRT) in stage IB2 and IIA2 squamous cervical cancer (SCC) and investigate the value of apparent diffusion coefficient (ADC) in outcome evaluation of different treatment strategies in the patients.

**Methods:**

A total of 149 patients with IB2 and IIA2 SCC who underwent pretreatment MRI and DWI scan were included. Patients were treated with NAT + RH or CCRT. Clinical indices and pathological factors were recorded. The imaging indices were measured including tumor size and tumor ADC values. Intraclass correlation coefficient was employed to evaluate the consistency of the indices measured by two observers. ROC curves were used to evaluate the cutoff values of clinical and imaging indices. Kaplan–Meier and Cox proportional hazard model were used to analyze the independent factors of disease-free survival (DFS).

**Results:**

The median follow-up period was 42.3 months. SCC-Ag, ADCmax and ADCmin were independent factors for DFS in the entire cohort. SCC-Ag, ADCmin and vascular invasion were independent factors for DFS in NAT + RH group. ADCmax and ADCmin were independent factors for DFS in CCRT group. ADCmin was the strongest independent factor for DFS in NAT + RH group, while ADCmax was that in CCRT group.

**Conclusion:**

The NAT + RH patients had similar DFS to that of CCRT in IB2 and IIA2 SCC, which could be a potential feasible alternative treatment. ADCmin and ADCmax were more valuable in evaluating the outcome of patients who underwent NAT + RH or CCRT, respectively.

## Key points

- DFS showed no significant difference between NAT + RH and CCRT patients.
- DWI-derived ADCmin and ADCmax contributed to evaluate DFS of NAT + RH and CCRT patients.
- ADCmin was a better predictor for NT + RH patients DFS while ADCmax was better for CCRT patients with IB2 and IIA2 SCC.

## Introduction

According to global cancer statistics in 2018, cervical cancer ranks as the fourth common cancer in the world and the fourth common cause of cancer death in women [1]. Previous studies showed that tumor volume was an unfavorable prognostic factor for patients with locally advanced cervical cancer (LACC) regardless of treatments [2]. The diameter of primary tumor > 4 cm was one of the recurrent risk factors, and the 5-year survival and disease-free survival (DFS) in patients with stage IB-IIA disease with diameter of tumors > 4 cm after surgery or radiotherapy were lower than those with diameter of tumors ≤ 4 cm [3]. According to NCCN, the options for IB2 and IIA2 disease are as follows: (1) concurrent chemoradiation (CCRT), (2) primary hysterectomy, (3) adjuvant hysterectomy after CCRT, and the first two treatments are adopted by most countries [4, 5]. Definitive CCRT may lead to adverse effects such as ovarian function damage, cystitis, proctitis or hematological toxicity; however, surgery continues to be the major treatment for IB2 and IIA2 disease in some countries or regions suffered from limitation of the radiation equipment and technology. The tumors > 4 cm increase the difficulty of the operation, and thus, the patients with IB2 and IIA2 disease need a comprehensive therapeutic strategy [6].

There were some studies that investigated the efficacy of neoadjuvant chemotherapy (NAC) and neoadjuvant radiotherapy followed by radical hysterectomy; however, there were some discrepancies in these studies [7–9]. Ouyang et al. [7] found that the patients who treated with neoadjuvant CCRT plus surgery had a worse 5-year disease-free survival (DFS) and overall survival (OS) than those who treated with primary operation in IB2 and IIA2 cervical adenocarcinoma. Ma et al. [8] found that there was no significant difference in survival between the patients with stage IB2 and IIA cervical cancer who received preoperative brachytherapy and chemotherapy followed by radical surgery and those who treated with chemoradiation, but the former had more favorable side effect profile. Another study by Zhang et al. [9] demonstrated that compared with the operation, preoperative intracavitary radiotherapy plus operation led to improvements in locoregional control rates with a similar incidence of complications.

As the quantitative parameter of DWI, apparent diffusion coefficient (ADC) has been used to predict the effect of CCRT in the patients with cervical cancer [10]. Some previous studies reported that LACC with lower ADC values of primary tumor was associated with recurrence and had worse prognosis after CCRT [11–13]. In contrast, other studies suggested that patients with lower ADC values of primary tumors had better prognostic outcome [14, 15]. However, the above studies only examined small cohorts of patients, and some of the aforementioned studies included both early and advanced disease. The differences of the tumor biological behavior between early and advanced cervical cancer might influence ADC values; moreover, the same CCRT regimen might have different effects on early and advanced disease. Thus, the relationship between ADC values and prognostic outcome may be affected, leading to disagreement of the results among different studies. Most of previous studies have appraised the effect of ADCs in evaluating the prognosis of patients treated with CCRT; however, there are few studies to analyzing those in evaluating the long-term prognostic outcome of patients treated with neoadjuvant therapy followed by radical hysterectomy (NAT + RH).

The squamous cell carcinoma is the most common histologic subtype of cervical cancer [16]. The previous study revealed that there was significant difference for progression-free survival between patients with squamous cell carcinoma and those with adenocarcinoma/others [10]. Moreover, the ADC values of the cervical cancers were different in different pathological types [17]. The purpose of this study was to investigate the effect of NAT + RH and CCRT on the prognosis of patients with stage IB2 and IIA2 squamous cervical cancer (SCC), and the value of ADC in outcome evaluation of different treatment strategies in the patients with IB2 and IIA2 SCC.

## Materials and methods

### Patients

The retrospective study was approved by the Institutional Review Board of our institution, the authorization number was 20210825YG, and the need for individual consent was waived by the committee. This study included the patients diagnosed as cervical cancer by biopsy in our hospital between February 2014 to February 2018, who met following criteria: (1) Patients were identified as stage IB2 or IIA2 according to FIGO 2009; (2) the initial treatment was neoadjuvant therapy (NAT included neoadjuvant radiotherapy, chemotherapy or chemoradiotherapy) combined with radical hysterectomy and pelvic lymph node dissection ± para-aortic node dissection or CCRT; (3) pretreatment MRI with DWI was performed and no treatment for cervical cancer was performed before MRI examinations; and (4) postoperative pathological report of neoadjuvant therapy had complete histopathological information. The exclusion criteria were as follows: (1) The images could not be evaluated due to artifacts or other factors; (2) patients with simultaneous malignancies; (3) the lesion was diagnosed as other histologic types except squamous cell carcinoma by biopsy reports; and (4) the patients could not complete standard course of CCRT.

**Fig. 1:**
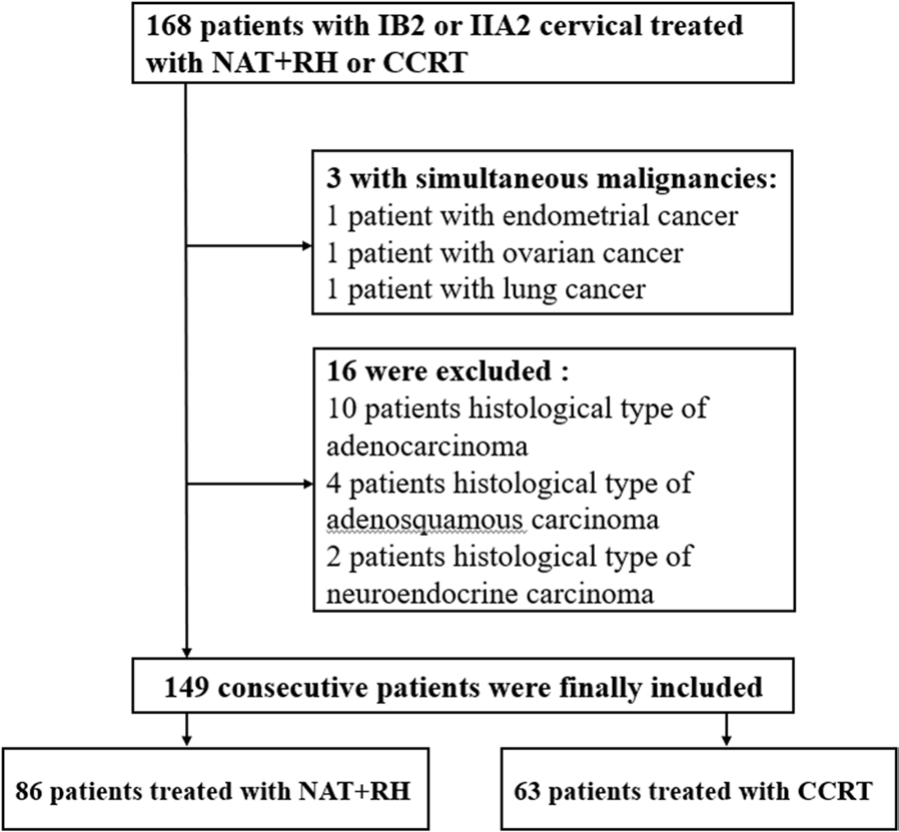
Flowchart of patient inclusion and exclusion. NAT + RH, neoadjuvant therapy followed by radical hysterectomy; CCRT, concurrent chemoradiation

A total of 168 patients were initially included, 19 were excluded, 149 consecutive patients were finally included in this study, 86 patients received NAT + RH, and 63 patients received CCRT. (Fig. 1). The mean age of the entire cohort was 50.9 ± 8.4 (range 26–67 years). Since the FIGO Cancer Report 2018 was published in October, the patients were staged according to the FIGO 2009 in this study. There were 62 patients with stage IB2, 37 patients received NAT + RH, and 25 patients received CCRT; there were 87 patients with stage IIA2, 49 patients received NAT + RH, and 38 patients received CCRT.

The pretreatment clinical indices were analyzed including age, FIGO stage, hemoglobin (normal range 110–160 g/L), squamous cell carcinoma antigen (SCC-Ag). Additionally, the operation methods (RH with pelvic lymphadenectomy or RH with pelvic + para-aortic lymphadenectomy) and lymph node status, vascular invasion and deep stromal invasion according to postoperative pathological reports were also analyzed in NAT + RH group.

### MRI protocol

All MR examinations were performed using a 3.0-T unit (Magnetom Trio; Siemens Medical Solutions, Germany) with an 8-channel phased array body coil and respiratory gating technology. Table 1 shows the parameters of MRI scanning.

**Table 1:** The parameters of MRI scanning

Sequence	Plane	TR/TE	Slice thickness (mm)	Gap (mm)	Matrix	Fov (cm)	Average	*b*-values (s/mm^2^)
FSE T1w	Axial	514/11	5	2	512 × 640	35	2	
FS FSE T2w	Axial	3000/106	5	2	294 × 448	35	2	
FSE T2w	Sagittal	3800/116	4	0.8	396 × 448	25	1	
FS FSE T1w	Axial	677/11	5	2	432 × 640	26	1	
FS FSE T1w	Coronal	590/11	5	1.25	544 × 640	26	1	
DWI(SS-EPI)	Axial	3500/93	4	0	256 × 320	27	4	0, 1000

*T1w* T1-weighted; *T2w* T2-weighted; *FS* fat saturation; *FSE* fast spin-echo; *TR* time of repetition; *TE* echo time; *Fov* field of view; *SS-EPI* single-shot echo-planar imaging

### Image analysis

A fellow (with 4 years of experience in pelvic MR imaging) and a radiologist (with 20 years of experience in pelvic MR imaging) measured and evaluated MR images independently, who were blinded to the clinical and pathological information of the patients.

Tumor size: The diameters of the tumor were measured in axial (transverse diameter) and sagittal (vertical and anteroposterior diameter) planes in T2W images, and the maximum diameter of the tumor (MDT) was recorded.

Positive lymph nodes in CCRT group were defined as: (a) The lymph nodes were observed in T2W images, short diameter ≥ 10 mm or short diameter between 8 and 10 mm with the ratio of short to long diameter ≥ 0.8 (identified as a round node) [18]; (b) a node that short diameter < 10 mm but with necrosis or extracapsular spread, necrosis showed heterogeneous signal in T2W and enhanced images, extracapsular spread showed irregular enhancement of lymph node capsule and infiltration of adjacent fat [19].

**Fig. 2:**
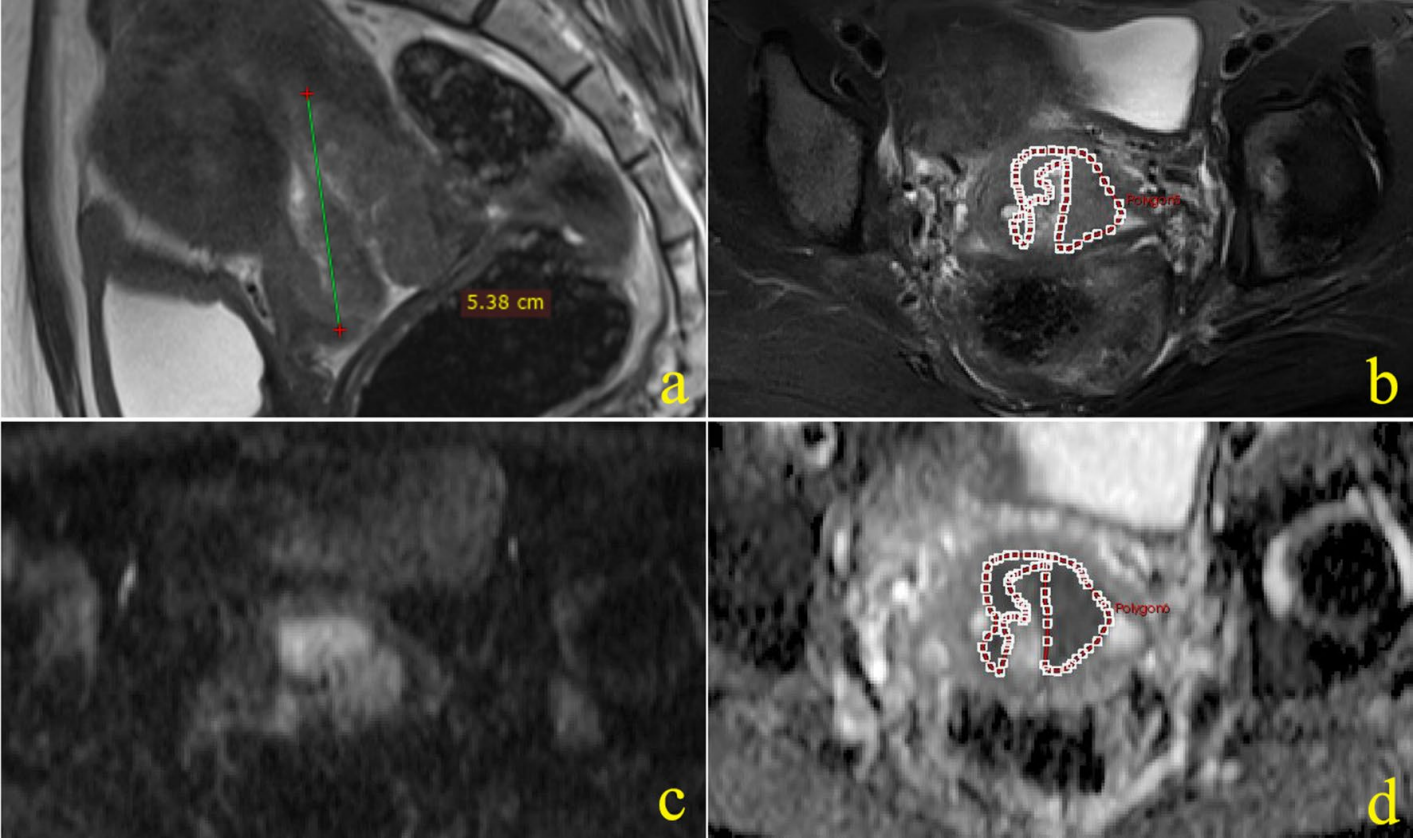
Images of a 45-year-old cervical cancer patient, (**a**) sagittal T2-weighted image of the tumor, the maximum diameter of tumor showed at sagittal image was 53.8 mm; **b**–**d** showed manually drew ROI at the axial largest slice of the tumor on different sequences, (**b**) axial fat suppressed T2-weighted FSE sequence; **c** axial DWI (*b* = 1000 s/mm^2^) sequence; **d** ROI of tumor on the corresponding slice of ADC map. FSE, fast spin-echo; DWI, diffusion-weighted imaging; ADC, apparent diffusion coefficient; ROI, regions of interest

Measurement of the ADC values of primary tumor: ADC maps were imported into a prototype software program (Omni Kinetics, GE Healthcare). The location of the lesions was observed by referring to T2W and DWI. Without knowing the clinical results, the radiologic fellow manually drew irregular shape of the regions of interest (ROIs) along the contour on each transverse section of the primary tumors. The ROI encompassed lesion areas as much as possible while certain interspaces to the margin were kept, avoiding any cysts and obvious necrotic areas. The cysts and necrotic areas appear as hyperintensity signal on T2W image, no enhanced on contrast-enhanced T1WI and bright area on ADC maps. The ROIs were manually drawn on the T2W images, and those obvious necrosis areas were excluded by carefully delineating ROIs along the solid part of lesions; then ROIs were manually drawn on ADC maps by referring the corresponding T2W ROIs and DWI sequence, trying to keep the ADC ROIs consist with T2W ROIs (Fig. 2). The ROIs from all slices of a tumor were finally merged as a three-dimensional volume of interest (VOI) of the whole tumor. The ADCmax and ADCmin values of the tumor were automatic identified from all included voxels by the Omni Kinetics software and then averaged mean of all voxels ADC values as the ADCmean value [20]. The ADCmax, ADCmean and ADCmin from VOI of the tumor were recorded.

The experienced radiologist measured MDT and ADC values according to above methods. The lymph node necrosis and extracapsular spread were determined in consensus.

### Treatments and follow-up

The patients in NAT + RH group were composed of neoadjuvant radiotherapy, chemotherapy and chemoradiotherapy by referring the previous studies [7, 21]. The patients received cisplatin-based or carboplatin-based chemotherapy regimens for 2 cycles. The regimens were paclitaxel + cisplatin (paclitaxel 135/m^2^, cisplatin 50 mg/m^2^), docetaxel + cisplatin (docetaxel 75 mg/m^2^), paclitaxel + carboplatin (area under the plasma concentration–time curve (AUC) = 5), docetaxel + carboplatin. Radiotherapy involved external beam radiotherapy (EBRT) or intracavity brachytherapy (ICBT). The EBRT (dose was about 35–45 Gy) or ICBT (A point Dt: 10–30 Gy) were performed as the preoperative radiotherapy regimens. The radical hysterectomy and pelvic ± para-aortic lymphadenectomy were performed within 3 weeks after the completion of NAT. The adjuvant radiotherapy (45–50 Gy EBRT in 25–30 fractions) and/or chemotherapy (initial regimens) were chosen to performed according to the pathological results of the patients. The patients received adjuvant CCRT who had lymph node metastases or had both vascular invasion and deep stromal invasion. The patients received adjuvant radiotherapy or chemotherapy who had only vascular invasion or deep stromal invasion.

The patients in CCRT group received radiotherapy combined with chemotherapy. The radiotherapy included EBRT (45-50 Gy, 1.8–2.0 Gy per fraction) and ICBT (A point Dt: 35–45 Gy). Three or four cycles chemotherapy regimens same as those in NAT + RH group were delivered concomitant with radiotherapy.

Patients were followed up every 3–6 months in the first 2 years, once every 6–12 months in next 3–5 years, and once a year after 5 years. The gynecological physical examination, pelvic MRI, chest and abdominal CT and SCC-Ag were performed in every follow-up visit. The time of locoregional recurrence and metastasis was recorded. DFS was calculated from the first day of receiving treatment to any locoregional recurrence or metastasis (within pelvic or distant) or the last follow-up.

### Statistical analysis

Statistical analysis was performed by using SPSS 17.0. Intraclass correlation coefficient (ICC) was employed to evaluate the interobserver agreement of the MDT and ADC values measured by the two observers. The evaluation criterion of reliability as follows: excellent, 1 ≥ ICC ≥ 0.75; fair to good, 0.75 > ICC ≥ 0.40; and poor, ICC < 0.40 [22]. The indices with ICC ≥ 0.75 were calculated average values. Chi-square test and Student t test were employed to compare the differences in clinical and imaging indices between NAT + RH and CCRT groups. Kruskal–Wallis test and Chi-square test were used for comparing the differences in clinical and imaging indices among three treatment modalities in NAT + RH group. The receiver operating characteristic curve was employed to calculate cutoff values according to the maximum Youden index for clinical and imaging consecutive variables. The 3-year DFS and related univariate were analyzed (including clinical and imaging variables) by using Kaplan–Meier and log-rank test. The Cox proportional hazard regression model was used for multivariate analyses to select independent risk factors. The variables were included which were statistically significant in the univariate analyses. *p* < 0.05 was considered significant in all statistical analyses.

## Results

### Agreement of MRI indices

The ICC of MDT measured by two observers was 0.871 (0.8108–0.895). The ICC of pretreatment ADCmax, ADCmean and ADCmin were 0.776 (0.676–0.861), 0.774 (0.704–0.828) and 0.845 (0.792–0.887), respectively.

### Comparison of evaluating indices among different groups and subgroups

There were no significant differences in clinical characteristics, imaging indices among the three neoadjuvant treatment modalities in the NAT + RH group (all *p* > 0.05) (Table 2). Table 3 shows that there were no significant differences in clinical or imaging indices between the NAT + RH group and CCRT group (all *p* > 0.05).

**Table 2:** Comparison of clinical indices, histopathologic factors and imaging indices among three different neoadjuvant therapy modalities followed by radical hysterectomy

Index	NAC + RH group (*n* = 31)	NAR + RH group (*n* = 28)	NACR + RH group (*n* = 27)	*p* value
Age	49.0 ± 9.1	51.1 ± 8.5	50.8 ± 8.6	0.601
FIGO				0.359
IB2	11	15	11	
IIA2	20	13	16	
Pretreatment hemoglobin				0.965
Normal	24	21	21	
Abnormal	7	7	6	
SCC-Ag	3.8 (1.3, 9.3)	3.1 (2.5, 4.9)	2.5 (1.1, 8.8)	0.766
ADCmax	1.102 ± 0.114	1.099 ± 0.145	1.097 ± 0.143	0.954
ADCmean	0.926 ± 0.123	0.897 ± 0.139	0.900 ± 0.141	0.622
ADCmin	0.783 ± 0.111	0.761 ± 0.112	0.760 ± 0.123	0.575
LNM				0.077
Positive	5	4	10	
Negative	26	24	17	
Vascular invasion				0.745
Positive	7	5	4	
Negative	24	23	23	
Deep stromal invasion				0.765
Positive	12	10	8	
Negative	19	18	19	
Operation method				0.460
Pelvic lymphadenectomy	21	21	16	
Pelvic + para-aortic lymphadenectomy	10	7	11	

*NAC + RH* neoadjuvant chemotherapy followed by radical hysterectomy; *NAR + RH* neoadjuvant radiotherapy followed by radical hysterectomy; *NACR + RH* neoadjuvant chemoradiotherapy followed by radical hysterectomy; *MDT* maximum tumor diameter; *SCC-Ag* squamous cell carcinoma antigen

**Table 3:** Comparison of clinical indices, histopathologic factors and imaging indices between neoadjuvant therapy followed by radical hysterectomy group and concurrent chemoradiotherapy group

Index	NAT + RH group (*n* = 86)	CCRT group (*n* = 63)	*p* value
Age	50.3 ± 8.7	51.9 ± 8.0	0.244
FIGO			0.683
IB2	37	25	
IIA2	49	38	
Pretreatment hemoglobin			0.537
Normal	66	51	
Abnormal	20	12	
SCC-Ag	3.1 (1.7, 6.0)	4.9 (1.8, 10.9)	0.099
MDT	45.0 (42.0, 50.0)	44.0 (41.0, 48.0)	0.277
ADCmax	1.080 ± 0.132	1.053 ± 0.117	0.205
ADCmean	0.908 ± 0.133	0.886 ± 0.117	0.296
ADCmin	0.769 ± 0.115	0.752 ± 0.117	0.379
LNM			0.053
Positive	19	23	
Negative	67	40	

*NAT + RH* Neoadjuvant therapy followed by radical hysterectomy; *CCRT* Concurrent chemoradiation; *SCC-Ag* Squamous cell carcinoma antigen; *MDT* Maximum tumor diameter; *LNM* Lymph node metastasis; MDT is in unit of mm, SCC-Ag is in unit of ng/ml

### Follow-up result

**Fig. 3:**
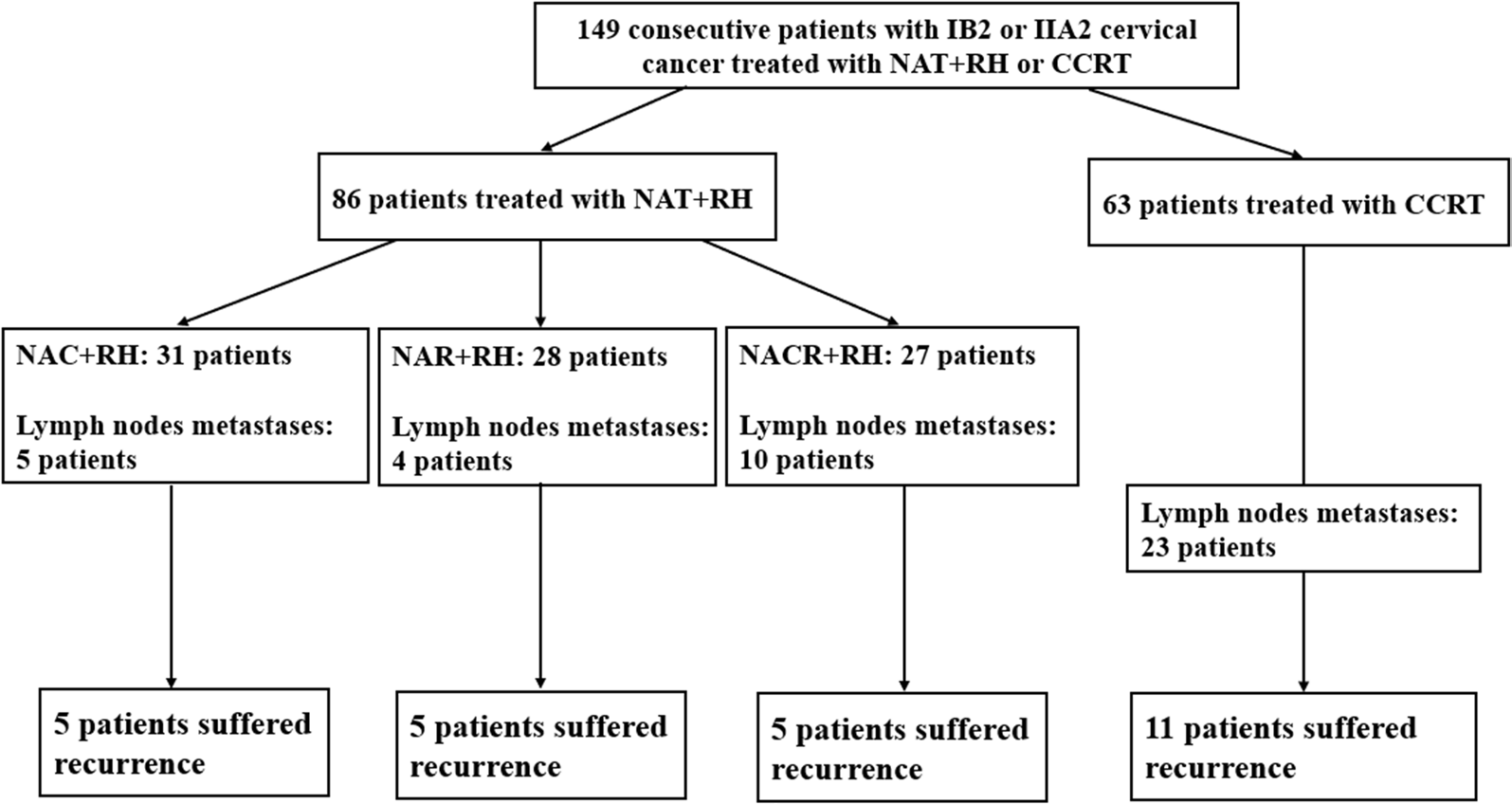
Flowchart of patients received the various treatment options, lymph node metastasis, and recurrence**.** NAT + RH, neoadjuvant therapy followed by radical hysterectomy; NAC, neoadjuvant chemotherapy; NAR, neoadjuvant radiotherapy; NACR, neoadjuvant chemoradiotherapy; CCRT, concurrent chemoradiation

As of the last follow-up visit, the median follow-up period of patients in entire cohort was 42.3 months (3.2–71.5 months), and 26 patients had recurrence. The median follow-up period of patients in NAT + RH group was 43.1 months (3.2–71.1 months) and that in CCRT group was 41.0 months (3.3–71.5 months). A total of 15 patients (17.4%) had recurrence in NAT + RH group: 6 of locoregional recurrence and 9 of metastases (6 of LNM, 2 of vaginal metastasis and 1 of distant metastases). Eleven patients (17.5%) had recurrence in CCRT group: 3 of locoregional recurrence and 8 of metastases (2 of LNM, 3 of vaginal metastasis and 3 of distant metastases) (Fig. 3).

**Fig. 4:**
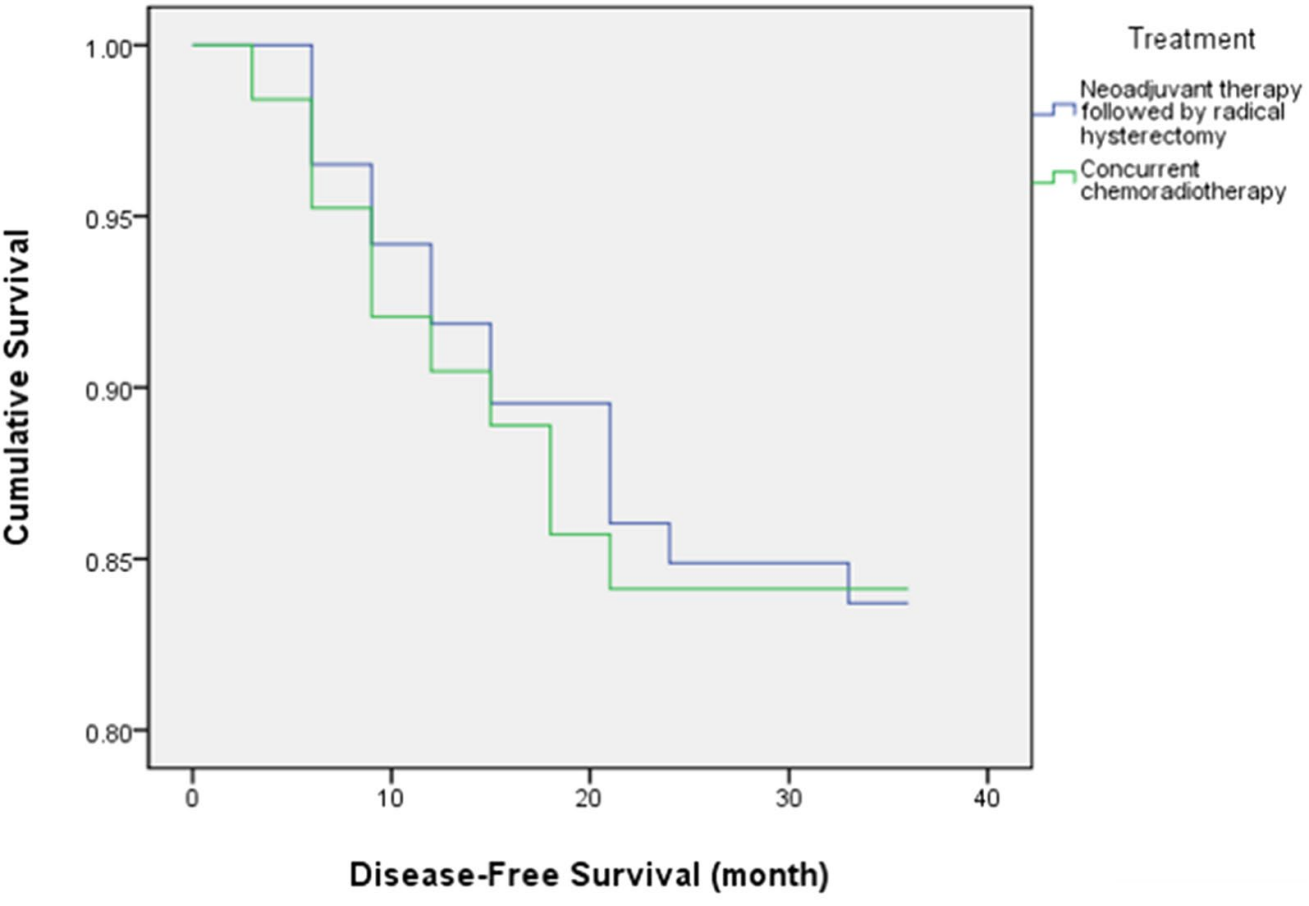
The DFS curves for NAT + RH and CCRT groups

The differences of 3-year DFS rate was not significant between NAT + RH and CCRT groups (*p* = 0.863) (Table 4, Fig. 4). In NAT + RH group, the differences of 3-year DFS rates were also not significant among three neoadjuvant treatments, between patients with or without adjuvant treatment, and among patients with three adjuvant treatment modalities (Table 4).

**Table 4:** Comparison of the 3-year DFS rate of different treatment modalities in NT group and between NT group and CCRT group

Index	Number	3-year DFS rate (%)	*p* value
Neoadjuvant treatment			0.967
NAC	31	84.0	
NAR	28	82.0	
NACR	27	78.0	
Adjuvant treatment			0.217
Adjuvant treatment	38	73.0	
Nonadjuvant treatment	48	88.0	
Adjuvant treatment			0.061
ACT	7	100.0	
ART	6	100.0	
ACRT	25	60.0	
Treatment			0.863
NT + RH	86	81.0	
CCRT	63	80.0	

*NAC* neoadjuvant chemotherapy; *NAR* neoadjuvant radiotherapy; *NACR* neoadjuvant chemoradiotherapy; *ACT* adjuvant chemotherapy; *ART* adjuvant radiotherapy; *ACRT* adjuvant chemoradiotherapy; *NAT + RH* neoadjuvant therapy followed by radical hysterectomy; *CCRT* concurrent chemoradiation

### Univariate survival analyses

**Fig. 5:**
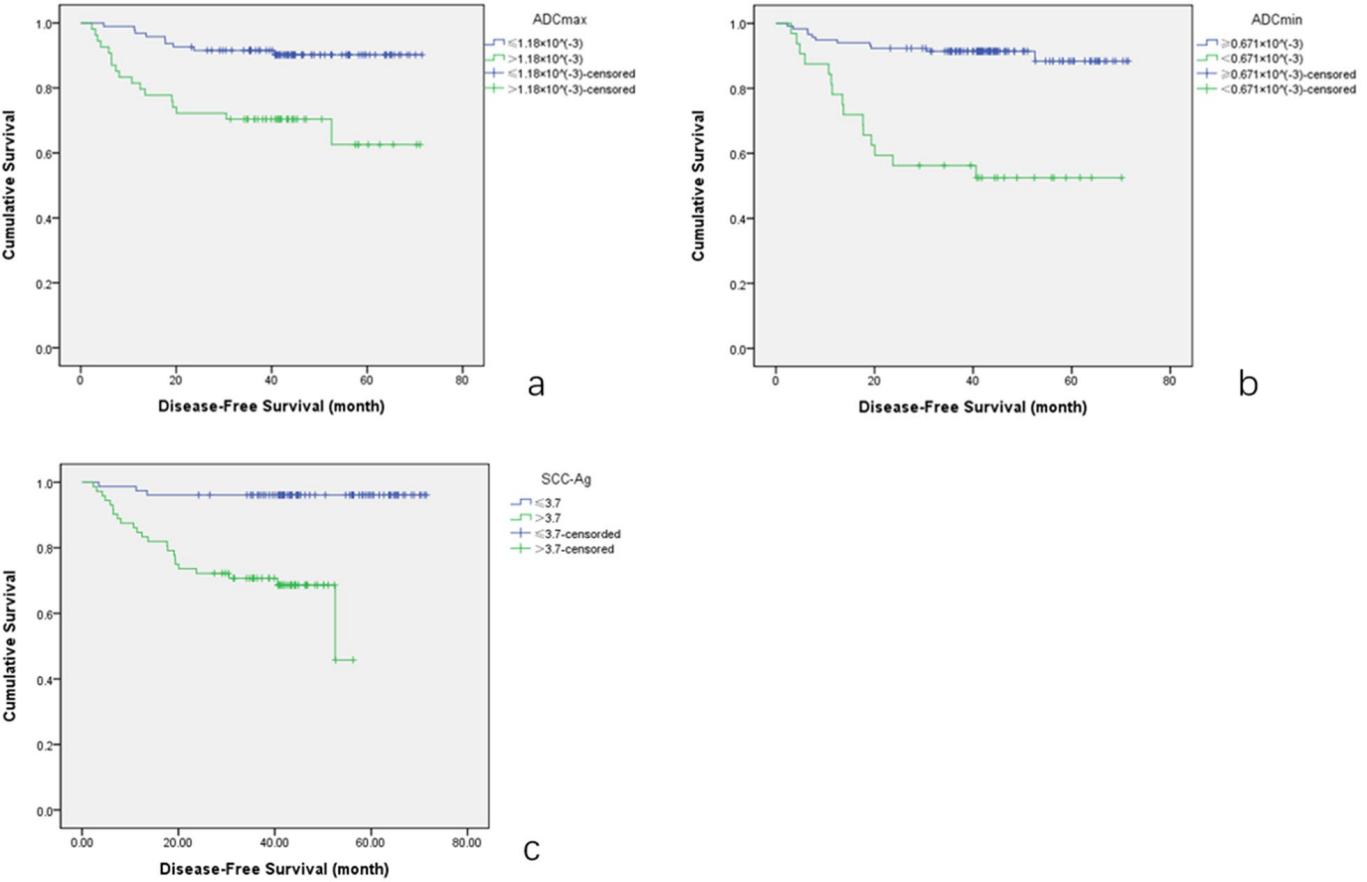
DFS curves of patients in the entire patient group; **a** ADCmax of the primary tumor; **b** ADCmin of the primary tumor; **c** pretreatment SCC-Ag of patients. DFS, disease-free survival; ADC, apparent diffusion coefficient; SCC-Ag, squamous cell carcinoma antigen

**Fig. 6:**
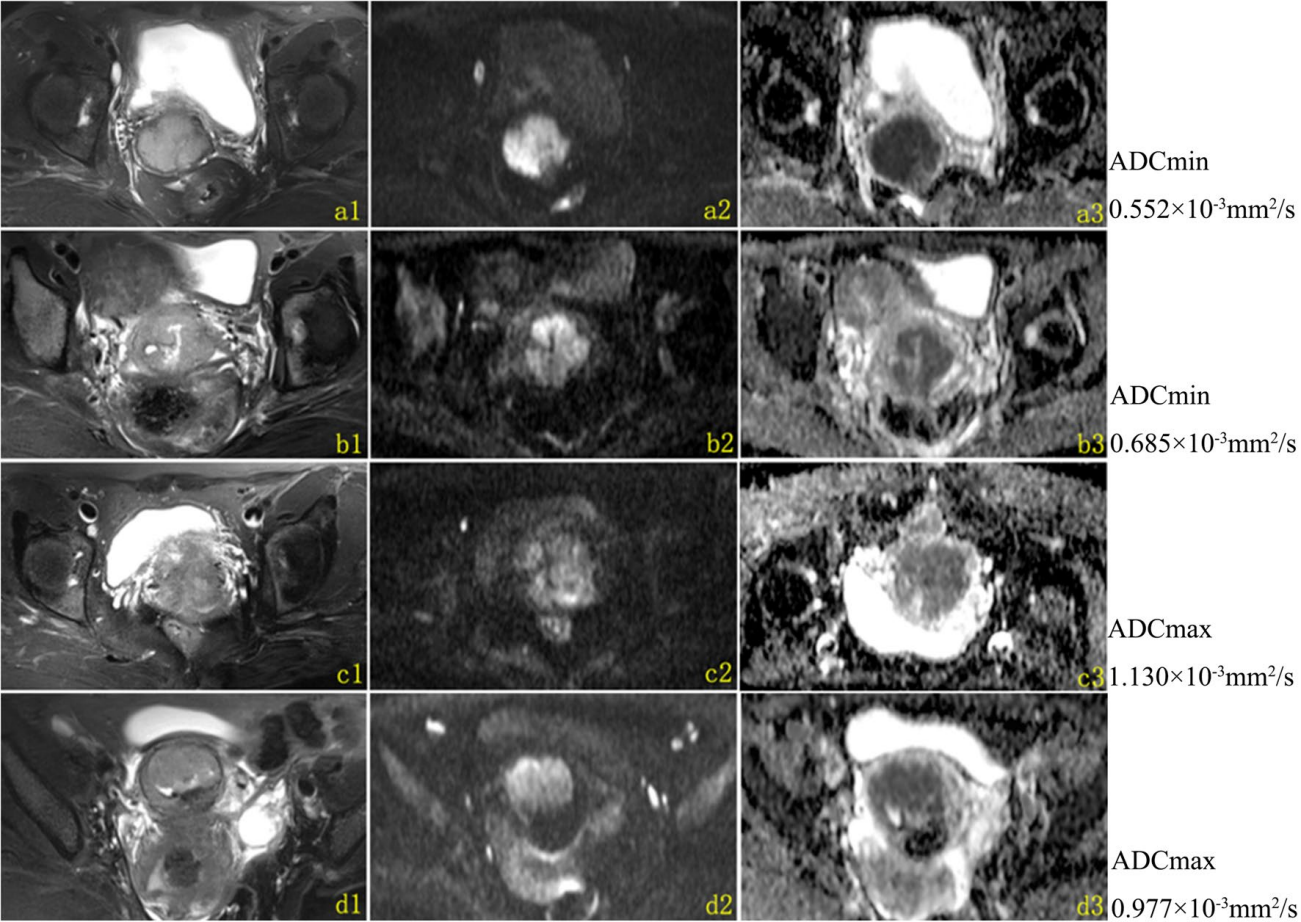
Different ADCmax and ADCmin values of lesions treated with NAT + RH (**a**–**b**) and CCRT (**c**–**d**); the maximum slice of the primary tumor was showed on T2W(a1-d1), DWI(a2-d2) and ADC(a3-d3) map. **a1**–**3** A 51-year-old patient treated with NAT + RH, the ADCmin of the primary tumor was 0.552 × 10^−3^mm^2^/s, DFS was 11.23 months; **b1**–**3** a 45-year-old patient treated with NAT + RH, the ADCmin of the primary tumor was 0.685 × 10^−3^mm^2^/s, DFS was 68.90 months; **c1**–**3** a 48-year-old patient treated with CCRT, the ADCmax of the primary tumor was 1.130 × 10^−3^mm^2^/s, DFS was 10.70 months; **d1**–**3** a 48-year-old patient treated with CCRT, the ADCmax of the primary tumor was 0.977 × 10^−3^mm^2^/s, DFS was 35.50 months**.** NAT + RH, neoadjuvant therapy followed by radical hysterectomy; CCRT, concurrent chemoradiation; DFS, disease-free survival

The univariate analyses of entire patient cohort, NAT + RH group and CCRT cohort are listed in Table 5. Figure 5 shows the DFS curves of patients in the entire patient group. Different ADCmax and ADCmin values of lesions treated with NAT + RH and CCRT are shown in Fig. 6.

**Table 5:** Univariate and multivariate analysis of clinical and imaging factors for evaluating disease-free survival

Variables	Univariate analysis		Multivariate analysis	
	Cutoff values	*p* value	Exp(B)(95%CI)	*p* value
*Entire cohort*				
ADCmax	1.118	** < 0.001**	19.0 (6.5–55.1)	** < 0.001**
ADCmin	0.671	** < 0.001**	25.8 (9.3–71.7)	** < 0.001**
SCC-Ag	3.7	**0.009**	2.9 (1.2–7.1)	**0.017**
MDT	43.6	**0.031**		0.159
LNM		**0.001**		0.180
Age	51.5	0.110		
FIGO stage		0.705		
Pretreatment hemoglobin		0.887		
ADCmean	0.753	> 0.05		
*NAT + RH group*				
ADCmin	0.671	** < 0.001**	6.2 (2.2–17.9)	**0.001**
SCC-Ag	4.7	** < 0.001**	5.0 (1.4–17.4)	**0.011**
Vascular invasion		**0.007**	4.2 (1.4–12.6)	**0.011**
Deep stromal invasion LNM		**0.026**		0.078
		**0.007**		0.342
Age	42.5	0.153		
FIGO stage		> 0.05		
Pretreatment hemoglobin		> 0.05		
ADCmax	1.118	> 0.05		
ADCmean	0.755	> 0.05		
Operation method		> 0.05		
MDT	42.5	0.212		
*CCRT group*				
ADCmax	1.113	** < 0.001**	11.2 (2.8–45.2)	**0.001**
ADCmin	0.649	** < 0.001**	5.5 (1.5–20.6)	**0.010**
MDT	47.8	**0.004**		0.059
LNM		**0.036**		0.732
Age	56.5	**0.021**		0.130
SCC-Ag	3.1	**0.020**		0.202
FIGO stage		0.289		
Pretreatment hemoglobin		0.973		
ADCmean	0.807	> 0.05		

Bold values mean the differences were statistically significant

*NAT + RH* neoadjuvant therapy followed by radical hysterectomy; *CCRT* concurrent chemoradiation; *SCC-Ag* squamous cell carcinoma antigen; *MDT* maximum tumor diameter; *LNM* lymph node metastasis; MDT is in unit of mm; ADC values are in units of 10^−3^mm^2^/s; and SCC-Ag is in unit of ng/ml

### Multivariate survival analyses

Multivariate analyses showed that in the entire patient group, ADCmax, ADCmin, SCC-Ag were independent prognostic factors for DFS of patients; ADCmin, SCC-Ag and vascular invasion were independent factors for DFS of patients in NAT + RH group; ADCmax and ADCmin were independent factors for DFS of patients in CCRT group (Table 5).

## Discussion

In this study, we found that there was no difference in 3-year DFS between patients received NAT + RH and CCRT. ADC values of the primary tumor could evaluate 3-year DFS of IB2 and IIA2 SCC, ADCmin was more valuable in evaluating 3-year DFS of patients underwent NAT + RH, while ADCmax was more valuable for the patients underwent CCRT. Lymph node metastasis was not the independent risk factor for 3-year DFS in this study.

There were contradictions among the results of prognostic outcome obtained from previous findings. Hsieh et al. reported that the 5-year DFS for the patients with IB2 disease who treated with NAC + surgery was similar to that for the patients received CCRT [23]. A randomized controlled trial including 633 patients with IB2-IIB cervical cancer demonstrated that the 5-year DFS of patients treated with NAC + surgery was lower than that of patients underwent CCRT [24]. However, another study showed that the 5-year DFS and OS of patients with IB2-IIB disease who underwent CCRT were both worse than those of patients who received NAC + RH [25]. Different FIGO stages disease included may be the reason for the inconsistency between the outcome of neoadjuvant therapy and CCRT among aforementioned studies. Although surgery is still applied to patients with IIB disease in some areas, according to NCCN guidelines, stage II B patients are definite candidates for CCRT.

In the entire patient cohort and NAT + RH group, the pretreatment SCC-Ag level was an independent factor for DFS. Wu et al. reported that SCC-Ag > 6.2 ng/ml was an independent prognostic factor for relapse-free survival of the patients with I B2 and II A2 disease, which was similar to the present study [26]. In this study, the incidence rates of vascular invasion were the independent factor for DFS of the patients in NAT + RH group. These were also consistent with the findings in some previous studies [27, 28].

In the entire cohort and CCRT group, the 3-year DFS was significantly worse in patients with a primary tumor ADCmax value above the cutoff than those with lower ADCmax. The lesion with necrosis part exhibited higher ADC values according to the previous studies [29, 30]. It is speculated that ADCmax may be able to sensitively reflect intratumoral necrosis. Necrotic tumors frequently are hypoxic, and poorly perfused, leading to diminished sensitivity to chemotherapy and radiotherapy [31]. The obvious necrotic areas were avoided to be included in ROIs in this study; however, there may be “invisible necrosis” because of the large tumor, which can be accurately reflected by ADCmax. The 3-year DFS was significantly worse in patients with ADCmin below the cutoff than those with higher ADCmin in all analyses. Zhao B et al. [32] also reported that the CCRT patients with recurrence within 2 years had significantly lower pretreatment ADCmin, and ADCmin was an independent factor for DFS. Nakamura et al. [33] also found that the patients with lower ADCmin and higher SUVmax of primary tumor had worse DFS and OS, which was similar to this study. High density of tumor cells and the decrease of intercellular space exhibit low ADC values [34], and thus, ADCmin could more accurately reflect the active infiltration and proliferation of tumor cells, which could be associated with outcome.

A notable finding in this study was that ADCmin was the strongest independent risk factor in NAT + RH group, while ADCmax was that in CCRT group. This result indicated that the patients with low pretreatment tumor ADCmin might be the better candidates for the NAT + RH instead of CCRT if the ones also exhibit high ADCmax meanwhile. The multivariable analysis also showed that ADCmax was not an independent factor for NAT + RH group. It is speculated that it was mainly due to the different emphasis of two treatment modalities. As chemo drugs and radiation of CCRT run through the treatment, the tumor will shrink but can exist during the treatment period. The “invisible” necrosis of the primary tumor might have a lasting negative impact on the efficacy of CCRT via leading to hypoxia. However, surgery is the predominant treatment for NAT, and patients undergo surgery after receiving 1–2 cycles of chemo and radiation. The effect of necrosis and hypoxia of the primary tumor on outcome eliminates after resecting the whole lesion. The effect of necrosis on NAT may be much less than that on CCRT and leaded to that ADCmax was not independent factor for NAT group. The results of this study showed that ADCmax and ADCmin might be valuable for the evaluation of the worse DFS of patients.

In this study, tumor ADCmean value was not correlated with DFS. In contrast, Nakamura reported that ADCmean was prognostic factor for DFS of IB1-IIB patients [35]. While the treatment modality in these two studies was only radical hysterectomy, that in the current study was NAT + RH or CCRT. The number of patients in Nakamura study was relative limited, the MDT of approximately 76% patients was < 4 cm, and histological subtypes of the tumors were heterogenous. The above reasons may result in the discrepancy between this study and the previous studies. Furthermore, ADCmean denotes the average ADC value of the whole tumor, but cannot accurately reflect the locoregional necrosis and active cellular proliferation. For patients who receive NAT + RH and CCRT, the pretreatment ADCmax and ADCmin could be more acute in evaluating the treatment outcome.

LNM was a high-risk factor for posttreatment recurrence according to NCCN guidelines. LNM was associated with DFS in univariate analysis; however, it was not the independent factor for DFS in this study. Ouyang et al. analyzed 91 patients with IB2 and IIA2 cervical adenocarcinoma underwent neoadjuvant therapy plus surgery or direct operation and found that positive LNM was an independent factor of DFS, which was different from the result of the present study [7]. However, all the cases were adenocarcinoma in Ouyang’s study, while only SCC was included in this study. Previous studies showed that there were differences in impact of LNM on prognosis between adenocarcinoma and SCC [36, 37]. In addition, the follow-up period in Ouyang’s study was 5 years, and the shorter follow-up duration in this study may be another reason that LNM was not an independent factor for DFS. Zhou also reported that LNM was not the independent predictor for recurrence of IB-IIA cervical SCC, which was consistent with our study [38].

There were several limitations in our study. First, this study was a single-center retrospective study and included three neoadjuvant treatments, with a limited number of cases for each treatment. Though there were no significant differences in baseline of clinical, imaging characteristics or 3-year DFS rate among the three neoadjuvant treatments, multicenter large sample prospective clinical trials are still needed to confirm the results in the future. The patient enrollment in this study was finished before FIGO 2018 released, FIGO staging 2018 was not used in this study, and the further studies about FIGO 2018 patients need to be performed in the future. Second, the median follow-up period in this study was less than 5 years, and the OS of the patients was not evaluated. In the future, the results of DFS and OS should be followed up for more than 5 years to evaluate the outcome more accurately. Third, only tumor ADC values were employed in this study, and multimodalities imaging or the introduction of radiomics analysis will be more accurate for the evaluation of tumor heterogeneity.

## Conclusion

The present study revealed that the 3-year DFS for NAT + RH on patients with IB2 and IIA2 SCC was similar to that of CCRT, which could be a potential feasible alternative treatment for I B2 and II A2 disease. ADCmin and ADCmax were valuable in evaluating the outcome of patients who underwent NAT + RH and CCRT, ADCmin was a more important predictor for DFS of NAT + RH patients while ADCmax was better for CCRT patients.

## Data Availability

The data and/or analyzed during the current study are available from the corresponding author on reasonable request.

## Abbreviations

ADC: Apparent diffusion coefficient
CCRT: Concurrent chemoradiotherapy
DFS: Disease-free survival
DWI: Diffusion-weighted imaging
LNM: Lymph nodes metastases
MDT: Maximum diameter of the tumor
MRI: Magnetic resonance imaging
NAC: Neoadjuvant chemotherapy
NAT: Neoadjuvant therapy
OS: Overall survival
RH: Radical hysterectomy
ROC: Receiver operating characteristic
ROI: Region of interest
SCC: Squamous cervical cancer
SCC-Ag: Squamous cell carcinoma antigen
